# Parathyroid hormone-related protein induces fibronectin up-regulation in rat mesangial cells through reactive oxygen species/Src/EGFR signaling

**DOI:** 10.1042/BSR20182293

**Published:** 2019-04-26

**Authors:** Hong-Min Chen, Jia-Jia Dai, Rui Zhu, Fang-Fang Peng, Su-Zhen Wu, Hong Yu, Joan C. Krepinsky, Bai-Fang Zhang

**Affiliations:** 1 Department of Biochemistry and Hubei Provincial Key Laboratory of Developmentally Originated Disease, Wuhan University School of Basic Medical Sciences, Wuhan, P.R. China; 2 Gannan Medical University, Ganzhou, P.R. China; 3 Division of Nephrology, McMaster University, Hamilton, Ontario, Canada

**Keywords:** epidermal growth factor receptor, extracellular matrix, parathyroid hormone-related protein

## Abstract

Parathyroid hormone-related protein (PTHrP) is known to be up-regulated in both glomeruli and tubules in patients with diabetic kidney disease (DKD), but its role remains unclear. Previous studies show that PTHrP-induced hypertrophic response in mesangial cells (MCs) and epithelial-mesenchymal transition (EMT) in tubuloepithelial cells can be mediated by TGF-β1. In the present study, although long-term PHTrP (1–34) treatment increased the mRNA and protein level of TGF-β1 in primary rat MCs, fibronectin up-regulation occurred earlier, suggesting that fibronectin induction is independent of TGF-β1/Smad signaling. We thus evaluated the involvement of epidermal growth factor receptor (EGFR) signaling and found that nicotinamide adenine dinucleotide phosphate oxidase-derived reactive oxygen species mediates PTHrP (1–34)-induced Src kinase activation. Src phosphorylates EGFR at tyrosine 845 and then transactive EGFR. Subsequent PI3K activation mediates Akt and ERK1/2 activation. Akt and ERK1/2 discretely lead to excessive protein synthesis of fibronectin. Our study thus demonstrates the new role of PTHrP in fibronectin up-regulation for the first time in glomerular MCs. These data also provided new insights to guide development of therapy for glomerular sclerosis.

## Introduction

Parathyroid hormone-related protein (PTHrP) was first discovered as the factor responsible for humoral hypercalcemia of malignancy. Different from the endocrine regulator parathyroid hormone (PTH), PTHrP is produced and secreted from nonmalignant fetal and adult tissues and plays a critical role in the development and/or growth regulation of bone, heart, mammary glands, and other tissues [1,2]. In the adult kidney, PTHrP is abundant in the glomeruli, tubules, and intrarenal arterial tree [3,4]. PTHrP exerts a modulatory action on renal function including renal plasma flow, glomerular filtration rate, etc [5].

PTHrP is known to be up-regulated in both glomeruli and tubules in patients with diabetic kidney disease (DKD) [6], but its role remains unclear. Izquierdo et al. developed a transgenic mouse model characterized by PTHrP overexpression in the renal proximal tubule. They found that chronic PTHrP overexpression showed no morphologic or functional alterations in basal conditions. But in streptozotocin (STZ)-induced experimental DKD, PTHrP transgenic mouse developed increased renal hypertrophy, a higher urinary albumin excretion (UAE), and lower total plasma protein levels than control mice [7]. It has been reported that PTHrP might promote epithelial-mesenchymal transition (EMT) through interaction with vascular endothelial growth factor (VEGF), TGF-β1, and epidermal growth factor (EGF) in renal tubuloepithelial cells [8,9]. In glomerular mesangial cells (MCs), continuous expression of or incubation with PTHrP induces a proliferative effect (24 h) followed by hypertrophy at 72 h [10–12], and PTHrP-induced hypertrophic response is probably mediated by TGF-β1 [12]. Our previous study showed that PTHrP could induce fibronectin up-regulation in rat MCs [13]. However, whether PTHrP-induced extracellular matrix (ECM) up-regulation is mediated by TGF-β1 is still not clear. The present study found that PTHrP (1–34) peptide-induced fibronectin up-regulation in rat MCs could be independent of TGF-β/Smad signaling. PTHrP (1–34) exposure induced reactive oxygen species (ROS)-dependent, Src kinase-mediated epidermal growth factor receptor (EGFR) transactivation and subsequent Akt and ERK1/2 activation, which involve in fibronectin up-regulation. It could help to make certain the role of PTHrP in the accumulation of ECM and provide new thought for the therapeutic strategy of glomerular sclerosis.

## Materials and methods

### Cell culture and treatments

After removal of kidneys from two to four Sprague–Dawley rats, trim the perirenal fat in PBS in a petri dish and then quickly remove the capsule. Cut in half lengthwise and remove the medulla. Then mush through the 250, 106, and 75 µm sieve with PBS sequencially. Wash with cold PBS to collect all the glomeruli and digest with collagenase for 15 min at 37°C. Next, place glomeruli in 100 mm plates with 8 ml of low-glucose DMEM (5.6 mM glucose) containing 20% FBS (Invitrogen, Carlsbad, U.S.A.), change the medium twice per week, and then subculture cell when confluent. Freeze down at each passage after passage 2. Characterize MCs by RT-PCR for vimentin, keratin, and desmin. MCs are positive for vimentin, negative for keratin. MCs were cultured in low-glucose DMEM containing 20% FBS, 100 U/ml penicillin, and 100 μg/ml streptomycin at 37°C in a 5% CO_2_ atmosphere. All of the experiments were performed between passages 6 and 18. *In vitro* studies have established that the amino-terminal peptide fragments are sufficient for the actions of PTHrP, as PTHrP (1–34) and PTHrP (1–36) peptide display high-affinity receptor binding and efficient receptor activation [14]. Based on these data, we substituted 100 nM PTHrP (1–34) peptide (Bachem, Bubendorf, Swiss) for PTHrP. Pharmacologic inhibitors were added at the indicated concentrations and durations before PTHrP (1–34) treatment: SB431542 (Tocris Bioscience, Bristol, U.K.), 5 µM for 30 min; H-89 (Selleck Chemicals, Houston, U.S.A.), 10 μM for 30 min; bisindolylmaleimide I (MedChem Express (MCE), Monmouth Junction, U.S.A.), 2 μM for 30 min; AG1478 (Tocris), 5 μM for 30 min; gefitinib (Tocris), 1 μM for 30 min; SU6656 (Sigma–Aldrich, St. Louis, U.S.A.), 10 μM for 30 min; protein phosphatase 1 (PP1, MCE), 10 μM for 30 min; N-acetylcysteine (NAC, Sigma), 10 μM for 10 min; apocynin (Sigma), 100 μM for 30 min; CRM197 (Sigma), 500 ng/ml for 60 min; GM6001 (MCE), 20 μM for 60 min; LY294002 (Sigma), 20 μM for 30 min; wortmannin (Sigma), 100 nM for 60 min; MK-2206 (Selleck), 1 μM for 60 min; U0126 (Sigma), 10 μM for 30 min.

### Quantitative real-time PCR

Quantitative PCR (qPCR) was performed using RNA extracted from rat MCs. Total RNA was isolated using RNA Extraction Kit (Qiagen, Germany). cDNA was reverse transcribed using Reverse Transcription kit (GeneCopoeia, Rockville, U.S.A.). Quantitative PCR was performed in duplicate using qPCR Kit (GeneCopoeia). Negative controls of cDNA were included for each gene set in all reactions to detect contamination. The primer sequences are shown as follows: GAPDH, sense, 5′- TGCACCACCAACTGCTTAGC-3′, antisense, 5′-GGCATGGACTGTGGTCATGAG-3′; TGF-β1, sense, 5′-AAACGGAAGCGCATCGAA-3′, antisense, 5′- GGGACTGGCGAGCCTTAGTT-3′. The thermo-cycle program was performed in MiniOpticon (Bio-Rad, Hercules, U.S.A.), and was set as 5 min at 95°C, followed by 30 cycles of at 95°C for 30 s, 60°C for 30 s, and 72°C for 1 min. Gene expression level was calculated using the ΔCt method relative to GAPDH.

### Protein extraction and western blotting

Rat MCs were lysed, and rat kidney cortices were homogenated in regular lysis buffer as described previously [15]. Protein concentration was determined by the Bradford’s method, and an equal amount of total protein were separated on 6% or 10% SDS-PAGE. For western blotting, proteins were transferred to nitrocellulose membranes (Merck Millipore, Darmstadt, Germany) for 2 h at 60 V. Membranes were then blocked with Tris buffer (pH 7.4) supplemented with 0.1% Tween-20 and 5% bovine serum albumin (BSA). The incubations with different primary antibodies were done in Tris buffer with 0.1% Tween-20 and 3% BSA overnight at 4°C. Primary antibodies included monoclonal TGF-β1 (1:500, Cell Signaling Technology (CST), Danvers, U.S.A.), monoclonal TβRII (1:1000, Santa Cruz Biotechnology, Santa Cruz, U.S.A.), monoclonal fibronectin antibody (1:2000, Merck), polyclonal phospho-EGFR-Y845 (1:1000), phospho-EGFR-Y1173 (1:1000), polyclonal EGFR antibody (1:1000), phospho-Akt-S473 (1:1000), polyclonal Akt antibody (1:1000), phospho-Src-Y416 (1:1000), polyclonal Src antibody (1:1000), polyclonal phospho-ERK1/2 antibody (1:1000), polyclonal ERK1/2 antibody, polyclonal phospho-Smad2/3 antibody (1:1000), polyclonal Smad2/3 antibody (1:1000, all CST), and polyclonal p47^phox^ antibody (1:1000, Santa Cruz). Monoclonal β-actin antibody (1:5000, Sigma) or GAPDH (1:1000, Santa Cruz) was used as a loading control. Next, membranes were washed using Tris buffer with 0.1% Tween-20 followed by incubating with secondary antibodies (1:10,000) for 1 h at room temperature and then washed and developed with ECL detection reagent (Amesham, Buckinghamshire, U.K.).

### Measurement of ROS generation by flow cytometry

MCs cultured in a 24-well plate were made quiescent in serum-free medium for 24 h and incubated with 5 μM 2′,7′-dichlorodihydrofluorescin diacetate (DCFH-DA, Beyotime Biotechnology, Shanghai, China) or dihydroethidium (DHE, Beyotime) at 37°C for 30 min, followed by washing three times with PBS. The cells were then left untreated or treated with 100 μM apocynin for 30 min or 10 μM NAC for 10 min before addition of 100 nM PTHrP (1–34) for 5 min, followed by washing three times with PBS. Next, the cells were trypsinized and resuspended in PBS. The fluorescence intensity (DCFH-DA: excitation wavelength 488 nm and emission wavelength 535 nm; DHE: excitation wavelength 300 nm and emission wavelength 610 nm) was measured by using flow cytometry (CytoFLEX, Beckman Coulter) and analyzed with FlowJo 7.6.1 software.

### Determination of nicotinamide adenine dinucleotide phosphate oxidase activity

Nicotinamide adenine dinucleotide phosphate (NADPH) oxidase (NOX) activity was determined by measuring the NADPH-dependent superoxide dismutase (SOD)-inhibitable cytochrome C reduction. The measurement was performed according to the manufacturers’ instruction (GENMED, Shanghai, China). Briefly, 900 μl of the reaction buffer containing NOX substrate (NADPH) and oxidized cytochrome C in a quartz cuvette was preincubated at 30°C for 3 min. Next, 100 μl supernatant from MC lysate of different groups was added to the reaction mixture and incubated at 30°C for 15 min. The absorbance at 550 nm was read by a spectrophotometer. The NOX activity was calculated as SOD-inhibitable cytochrome C reduction and expressed as O_2_^−^ in nmol/min/mg.

### Diabetic rat model

The Center for Animal Experiment in Wuhan University approved all experiments and all experiments were performed according to Chinese Ethics Community Guidelines. Male Sprague–Dawley rats (weighing 200–225 g) were injected with STZ by tail vein (55 mg/kg body weight, freshly prepared in 0.1 mol/l citrate buffer, pH 4.5) or vehicle alone to induce diabetes. Total 2 days later, hyperglycemia (blood glucose > 20 mM) was confirmed using a reflectance meter (One Touch, Lifescan, Milpetas, U.S.A.). Diabetic rats were injected with different dosages of PTHrP (1–34) peptide (Bachem) by subcutaneous (s.c.) injection (40, 80, or 160 μg/kg body weight, respectively). Injections were once daily for 5 days per week for 3 months [16]. Blood glucose levels were monitored and systolic blood pressure was determined weekly in all diabetic rats. Urine was collected for 24 h in metabolic cages at 3 months. Rats were then anesthetised for kidney removal. Kidney samples were rapidly excised, weighed, and frozen in liquid nitrogen. At the time of killing, renal hypertrophy was assessed by kidney to body weight ratio (mg/g).

### Biochemical analysis

Serum calcium and phosphate, serum creatinine, urinary creatinine, and albumin were determined by the clinical laboratory of Zhongnan Hospital, Wuhan University. Creatinine clearance, Ccr (ml·min^−1^·100 g body weight^−1^) was calculated as urine creatinine × urine volume (ml·min^−1^)/serum creatinine/100 g body weight.

### Immunohistochemistry

The renal cortex was fixed by 4% paraformaldehyde, embedded in paraffin and then cut into slices with a thickness of 4 μm. Next, renal cortical sections were routinely deparaffinized, rehydrated, and subjected to heat-induced antigen retrieval. After blocking endogenous peroxidase activity and rinsing, these sections were blocked with 5% BSA in PBS and incubated with mouse monoclonal fibronectin antibody (1:500, BD Biosciences, San Jose, U.S.A.) at 4°C overnight. The sections were subsequently incubated with anti-mouse secondary antibody for 1 h and then stained by 3, 3′-diaminobenzidine (DAB) to produce brown colorization. Hematoxylin was used for counterstaining. Negative control was performed without incubation within primary antibody. Positive staining signal percentage was quantitated using the image analysis system (Image-Pro Plus 7.0). Total ten high-power microscope fields (400×) were selected randomly.

### Statistical analysis

All data are presented as means ± S.E.M. Experimental repetition times (n) were given in figure legends. Results analysis was performed by one-way ANOVA with Turkey Honestly Significant Difference (HSD) for *post hoc* analysis (SPSS 20.0 for Windows). A *P* value <0.05 is considered statistical significant.

## Results

### PTHrP (1–34)-induced fibronectin up-regulation is independent of TGF-β1 signaling in rat MCs

**Figure 1:**
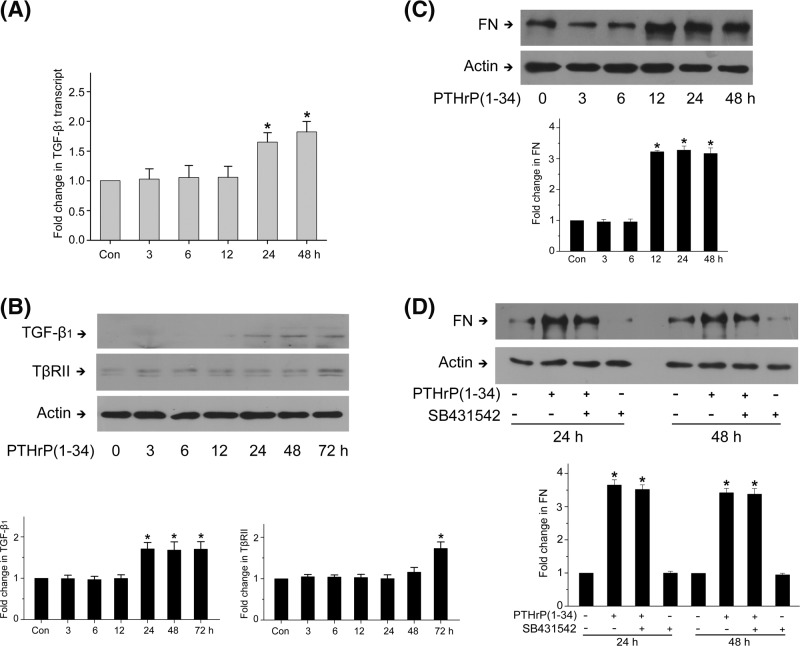
PTHrP (1–34)-induced fibronectin up-regulation is independent of TGF-β1 signaling MCs were treated with 100 nM PTHrP (1–34) for indicated time. (**A**) mRNA level of TGF-β1 was assessed by RT-PCR (*P<0.05 vs control, *n*=4). (**B**) Protein expression of TGF-β1 and TβRII was detected by western blot, with β-actin used as loading control (*P<0.05 vs control, *n*=3). (**C**) Protein level of fibronectin was assessed by western blot (*P<0.05 vs control, *n*=4). (**D**) MCs were pretreated with TGFβ receptor inhibitor SB431542 before PTHrP (1–34) incubation for 24 or 48 h. Protein level of fibronectin was assessed by western blot (*P<0.05 vs control, *n*=3).

It has been reported that prolonged PTHrP exposure induces a hypertrophic response mediated by TGF-β1 in cultured human MCs [12]. We further observed whether the TGF-β1 system involves in PTHrP-induced fibronectin up-regulation in rat MCs. PTHrP (1–34) increased the mRNA and protein expression of TGF-β1 after 24 h (Figure 1A,B), and an increase in TβRII protein expression was also induced at 72 h (Figure 1B), which is consistent with previous findings [12]. We then investigated the effect of PTHrP (1–34) exposure on fibronectin protein level. As shown in Figure 1C, fibronectin protein expression markedly increased from 12 h and continued to 48 h. However, Smad2/3 phosphorylation was not affected by PTHrP (1–34) until 72 h (Supplementary Figure S1A). We next used TGF-β receptor inhibitor SB431542 to block TGF-β1 signaling. It has no effect on PTHrP (1–34)-induced fibronectin up-regulation (Figure 1D). These results strongly indicate that PTHrP might induce fibronectin up-regulation independent of TGF-β1/Smad signaling.

### PTHrP (1–34) induces Src-dependent EGFR transactivation and Akt phosphorylation

Given the well-known effect of PKA and PKC as downstream effects of PTH/PTHrP signaling [17], we evaluated their involvement in PTHrP-induced fibronectin up-regulation by using the PKA inhibitor H-89 and the PKC inhibitor bisindolylmaleimide I. Neither inhibitor affected PTHrP (1–34)-induced FN up-regulation (Supplementary Figure S1B,C), indicating that PTHrP-induced fibronectin up-regulation is probably independent of PKA and PKC signaling.

**Figure 2:**
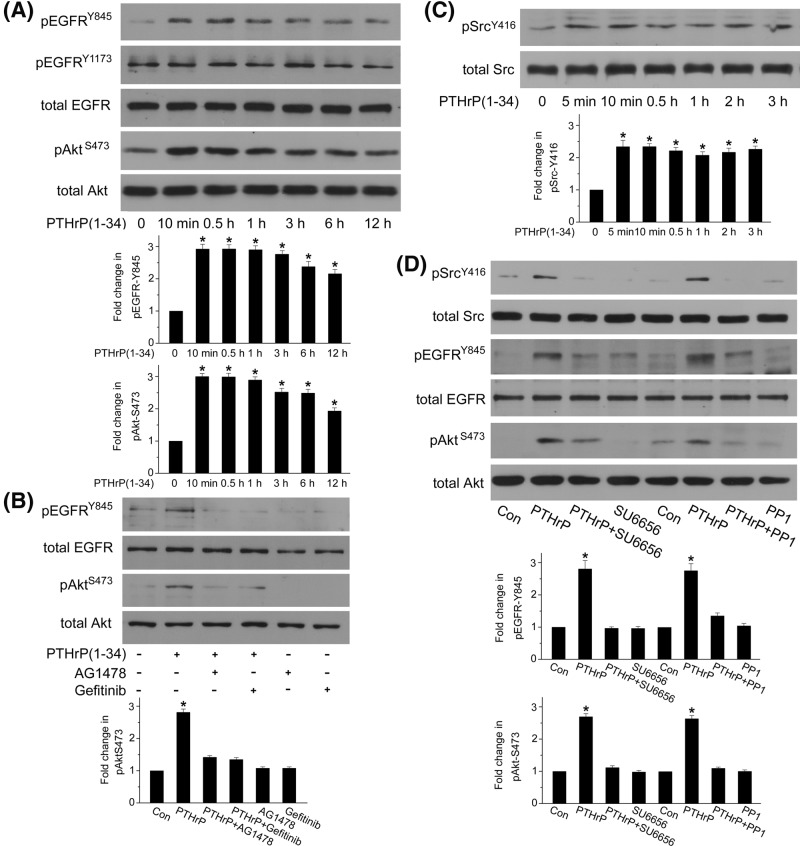
PTHrP-induced EGFR transactivation and Akt activation are mediated by Src kinase (**A**) MCs were treated with 100 nM PTHrP (1–34) for indicated period, and EGFR phosphorylation at Y845 and Akt phosphorylation at S473 were detected by western blot (*P<0.05 vs control, *n*=4). (**B**) MCs were pretreated with specific EGFR kinase inhibitor, AG1478 or Gefitinib, before PTHrP (1–34) incubation for 1 h. Akt phosphorylation at S473 was assessed by western blot (*P<0.05 vs control, *n*=3). (**C**) MCs were treated with 100 nM PTHrP (1–34) for indicated durations, and Src phosphorylation at Y416 was detected by western blot (*P<0.05 vs control, *n*=4). (**D**) MCs were pretreated with specific Src inhibitor, PP1, or SU6656, prior to PTHrP (1–34) treatment for 1 h. EGFR phosphorylation at Y845 and Akt phosphorylation at S473 were assessed by western blot (*P<0.05 vs control, *n*=4).

EGFR is known to aid in transmitting signals for diverse nonligand mediated stimuli in a process known as transactivation [18]. Stimulation of PTH type 1 receptor (PTH1R), common to PTH and PTHrP, leads to EGFR transactivation in human embryonic kidney cells HEK-293, murine osteoblasts and renal tubule cells [9,19,20]. We then tested whether the activation of PTH1R by PTHrP (1–34) treatment induced EGFR transactivation in rat MCs. As seen in Figure 2A, PTHrP (1–34) induced sustained EGFR transactivation, as determined by phosphorylation at tyrosine 845 (Y845), but not Y1173. PI3K/Akt pathway is usually the downstream of EGFR signaling, so we investigated whether PTHrP (1–34) induced Akt activation. As a result, Akt phosphorylation at serine 473 (S473), an indicator of Akt activity, was significantly increased at 10 min by PTHrP (1–34) treatment (Figure 2A). Moreover, Akt activation triggered by PTHrP (1–34) was prevented when we treated MCs with the specific EGFR kinase inhibitor gefitinib or AG1478 (Figure 2B), suggesting that EGFR activation is an important upstream event in PI3K/Akt pathway.

Several studies confirmed that PTHrP (107–111) peptide (known as osteostatin domain) may activate c-Src (Src) kinase in osteoblastic cells [21,22]. Whether PTHrP (1–34) peptide mediates Src kinase activation is not known. We thus examined the phosphorylation level of Src kinase at tyrosine 416 (Y416), which represents the activation of Src kinase [23]. In response to PTHrP (1–34), Src phosphorylation on Y416 increased at 5 min, and lasted until 3 h, the last time point tested (Figure 2C). The increase in phosphorylation level of Src is earlier than that of EGFR phosphorylation, indicating that Src kinase might mediate EGFR transactivation in MCs. We thus took advantage of specific Src kinase activity inhibitor PP1 and SU6656 to investigate the effects of Src kinase on EGFR transactivation and subsequent Akt activation, and found that Src kinase inhibition blocked PTHrP (1–34)-induced EGFR and Akt phosphorylation (Figure 2D). Taken together, these results suggested that PTHrP (1–34)-induced EGFR transactivation and Akt activation require Src kinase.

### ROS mediate PTHrP (1–34)-induced Src activation and EGFR/Akt phosphorylation

**Figure 3:**
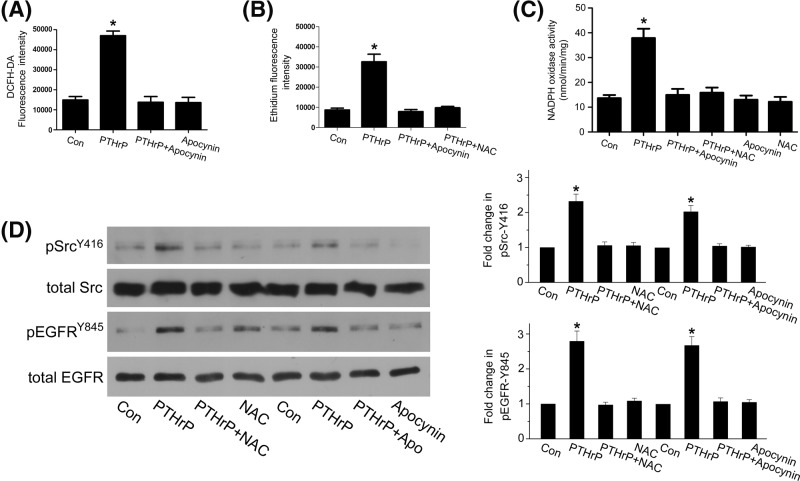
NOX-dependent ROS generation is required for Src activation and EGFR phosphorylation by PTHrP (1–34) **(A**) MCs were incubated with DCFH-DA at 37°C for 30 min and then treated or untreated with selective NOX inhibitor, apocynin, before 100 nM PTHrP (1–34) stimulation for 5 min, and H_2_O_2_ production was assayed by flow cytometry (*P<0.05 vs control, *n*=3). (**B**) MCs were incubated with DHE at 37°C for 30 min and then treated with apocynin or NAC before 100 nM PTHrP (1–34) stimulation for 5 min. Superoxide production was assayed by flow cytometry (*P<0.05 vs control, *n*=3). (**C**) MCs were treated or untreated with apocynin or NAC before 100 nM PTHrP (1–34) stimulation for 5 min, and NOX activity was assayed by measuring the NADPH-dependent superoxide dismutase (SOD)-inhibitable cytochrome c reduction (*P<0.05 vs control, *n*=3). (**D**) MCs were pretreated with NAC or apocynin before PTHrP (1–34) treatment for 1 h. Src phosphorylation at Y416 and EGFR phosphorylation at Y845 were assessed by western blot (*P<0.05 vs control, *n*=4).

It is known that NOX-derived ROS can mediate Src activation [24–26]. In rat MCs, PTHrP (1–34) increased hydrogen peroxide (H_2_O_2_) production as early as 5 min, which was inhibited by a selective NOX inhibitor, apocynin (Figure 3A, Supplementary Figure S2) and ROS inhibitor NAC also prevented PTHrP (1–34)-induced superoxide generation (Figure 3B, Supplementary Figure S3). To further verify the effect of PTHrP (1–34) on ROS, we tested NOX activity. As seen in Figure 3C, PTHrP (1–34) increased NOX activity at 5 min in MCs, which was inhibited by apocynin and NAC. Put together, these results indicated that PTHrP (1–34) induced NOX-dependent ROS generation. Next, we examined whether NOX-derived ROS mediates Src activation. Both apocynin and NAC abolished PTHrP (1–34)-induced Src phosphorylation at Y416 and EGFR phosphorylation at Y845 (Figure 3D), indicating that NOX-dependent ROS generation is essential for Src activation and EGFR phosphorylation.

**Figure 4:**
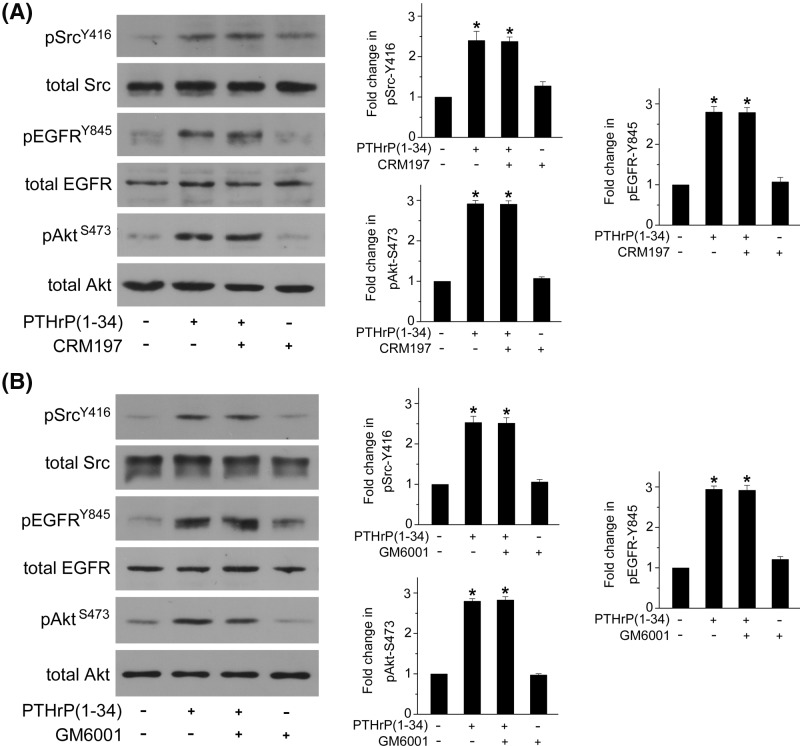
PTHrP (1–34)-induced Src activation and EGFR transactivation are independent of MMPs-mediated HB-EGF release MCs were treated with CRM197 (**A**), the HB-EGF inhibitor or GM6001 (**B**), a pan-specific MMPs inhibitor, prior to 100 nM PTHrP (1–34) incubation. The extent of phosphorylation of Src at Y416, EGFR at Y845, and Akt at S473 was detected by Western blot (*P<0.05 vs control, *n*=4).

On the other hand, G protein-coupled receptors (GPCRs), including PTH1R, are known to transactivate EGFR by soluble EGF-like ligand (such as heparin-binding EGF, HB-EGF) release and binding in a variety of cell types [9,19,20]. Although PTH (1–36) rapidly (within 5 min) and transiently induces EGFR transactivation through PKC and matrix metalloproteinases (MMPs)-mediated proteolytic processing of EGFR ligands in murine cortical tubule cells [9], either GM6001, a pan-specific MMPs inhibitor or CRM197, the HB-EGF inhibitor could not prevent Src, EGFR, and Akt phosphorylation by PTHrP (1–34) in rat MCs (Figure 4A,B), suggesting that PTHrP (1–34)-induced sustained Src activation and EGFR transactivation are independent of MMPs-mediated HB-EGF cleavage and release.

### ROS-mediated Src/EGFR/Akt signaling mediates PTHrP (1–34)-induced fibronectin up-regulation

**Figure 5:**
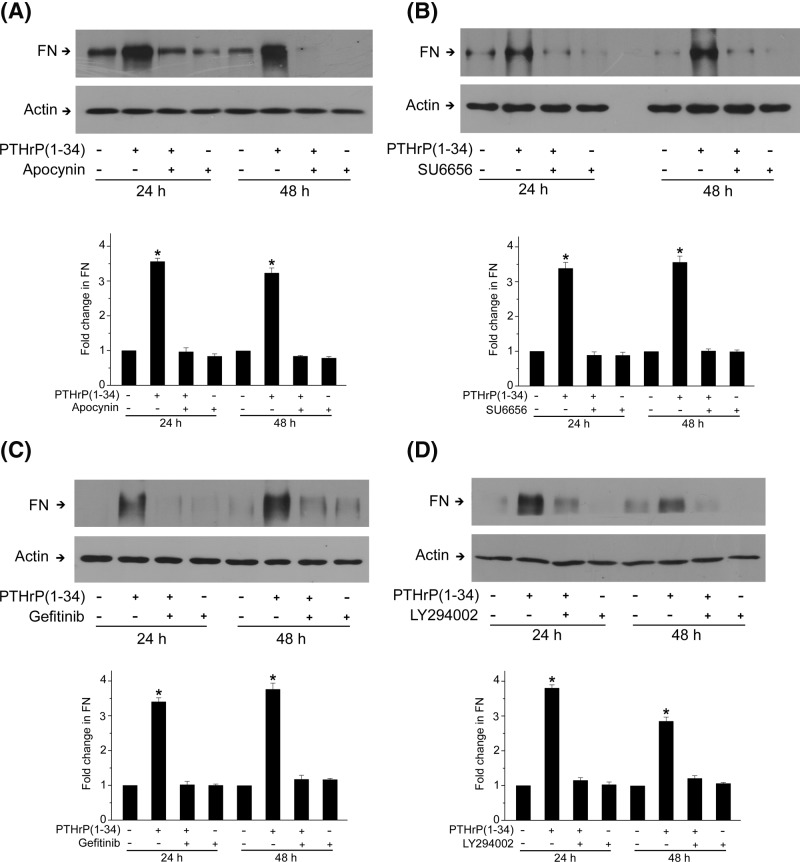
PTHrP (1–34)-induced fibronectin up-regulation in rat MCs is dependent on NOX and Src/EGFR/PI3K signaling MCs were pretreated with NOX inhibitor apocynin (**A**), Src inhibitor SU6656 (**B**), EGFR kinase inhibitor gefitinib (**C**), or PI3K inhibitor LY294002 (**D**) followed by incubation with 100 nM PTH (1–34) for 24 or 48 h. The protein level of fibronectin was assessed by western blot, with β-actin used as loading control (*P<0.05 vs control, *n*=5).

Next, we investigated whether ROS-mediated Src/EGFR/Akt signaling involves in PTHrP (1–34)-induced fibronectin up-regulation in rat MCs. Both NOX inhibitor and Src kinase inhibitor blocked fibronectin up-regulation in response to PTHrP (1–34) exposure (Figure 5A,B). Inhibition of EGFR signaling by gefitinib, or inhibition of Akt signaling with the specific PI3K inhibitor, LY294002, also prevented fibronectin up-regulation (Figure 5C,D). These results indicated that PTHrP (1–34)-induced fibronectin up-regulation is dependent on NOX-derived ROS in rat MCs, which activate Src kinase and downstream EGFR/PI3K/Akt signaling.

**Figure 6:**
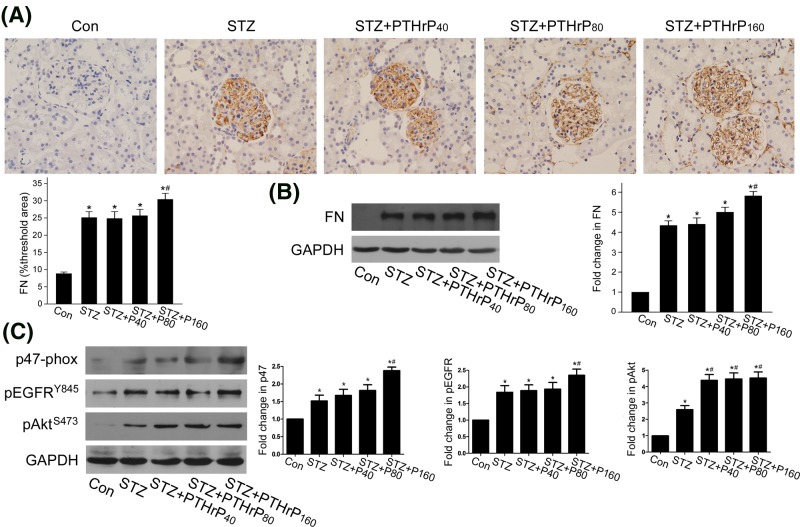
PTHrP (1–34) increased protein levels of p47^phox^, phosphor-EGFR, phosphor-Akt, and fibronectin in renal cortex of diabetic rats Diabetic SD rats were untreated or treated with PTHrP (1–34) by s.c. injection at 40, 80, or 160 μg/kg body weight for 3 months. (**A**) Representative microphotographs of fibronectin immunostaining (400 magnification) were shown. Computer-aided analysis of renal cortical fibronectin expressed as percent area (*P<0.05 vs control, ^#^*P*<0.05 vs STZ group, *n*=5). (**B**) The protein level of fibronectin in renal cortex was detected by western blot, with GAPDH used as loading control (*P<0.05 vs control, ^#^*P*<0.05 vs STZ group, *n*=8). (**C**) The protein levels of p47^phox^, phosphor-EGFR Y845, and phosphor-Akt S473 in renal cortex were detected by western blot, with GAPDH used as loading control (*P<0.05 vs control, ^#^*P*<0.05 vs STZ group, *n*=8).

To verify this, we further observed the effect of long-term PTHrP injection on STZ-induced diabetic rats. During the 3 months of PTHrP (1–34) treatment, there was no change in the serum calcium and phosphate levels amongst groups. Diabetic rats had remarkably higher UAE compared with controls, and this was unaffected by PTHrP (1–34). Diabetic rats developed renal hypertrophy, while administration of all three doses of PTHrP (1–34) aggravated renal hypertrophy (Table 1). Immunohistochemistry of cortical sections for fibronectin showed significantly increased staining both in the glomeruli and tubular cells in diabetic rats at 3 months compared with control. Fibronectin staining was stronger in 160 μg/kg PTHrP (1–34) group compared with diabetic rats (Figure 6A), indicating that long-term treatment with PTHrP promote fibronectin up-regulation in the renal cortices of diabetic rats. We also tested the protein level of fibronectin in renal cortex by western blot. Same as immunohistochemistry, increased fibronectin protein level was also observed in PTHrP (1–34)-injected diabetic rat kidney at 3 months (Figure 6B).

**Table 1:** Clinical characteristics of control rats and STZ-induced diabetic rats untreated or treated with PTHrP at 3 months

	Control	STZ	STZ+P40	STZ+P80	STZ+P160
N	9	8	8	8	8
Glucose (mmol/l)	3.99 ± 0.69	29.13 ± 1.08*	28.96 ± 1.64*	29.06 ± 1.35*	30.26 ± 1.75*
Systolic blood pressure (mmHg)	113.0 ± 1.6	117.0 ± 1.0	110.0 ± 0.8	119.0 ± 2.0	116.0 ± 2.1
Kidney/body weight (mg/g)	3.1 ± 0.10	5.4 ± 0.06*	5.8 ± 0.31*^#^	6.0 ± 0.17*^#^	6.4 ± 0.37*^#^
Ccr (ml/min)	4.5 ± 0.22	5.2 ± 0.38	5.3 ± 0.51	5.3 ± 0.49	5.1 ± 0.33
Albuminuria (mg/24 h)	11.66 ± 0.81	89.49 ± 4.78*	90.36 ± 3.00*	90.23 ± 5.73*	92.35 ± 5.13*
Serum calcium (mmol/l)	2.20 ± 0.05	2.13 ± 0.09	2.10 ± 0.19	2.25 ± 0.10	2.14 ± 0.18
Serum phosphate (mmol/l)	2.12 ± 0.16	1.94 ± 0.10	2.13 ± 0.27	2.03 ± 0.24	2.10 ± 0.45

Data are means ± S.E. *P<0.05 vs control. ^#^*P*<0.05 vs STZ group.

An important source of ROS in the diabetic glomeruli is NOX1 and NOX2 [27,28]. The cytosolic p47^phox^ subunit is a key regulator of NOX1 and NOX2 [29,30]. Since PTHrP (1–34) induced NOX-dependent ROS generation in rat MCs, we investigated the role of PTHrP (1–34) in the expression of p47^phox^. Increased p47^phox^ protein level was seen in diabetic renal cortex, and p47^phox^ protein level was even higher in 160 μg/kg PTHrP (1–34) group (Figure 6C). We also observed whether PTHrP (1–34) treatment has an effect on EGFR and Akt activation in diabetic rat kidney. Consistent with the *in vitro* findings, the phosphorylation levels of EGFR and Akt were markedly increased in renal cortex in 160 μg/kg PTHrP (1–34)-injected diabetic rats (Figure 6C). These results collectively suggested that PTHrP (1–34) induced ROS production and increased EGFR/Akt phosphorylation in diabetic rat kidney.

### Src- and EGFR/PI3K-dependent ERK1/2 activation also mediates PTHrP (1–34)-induced fibronectin up-regulation

**Figure 7:**
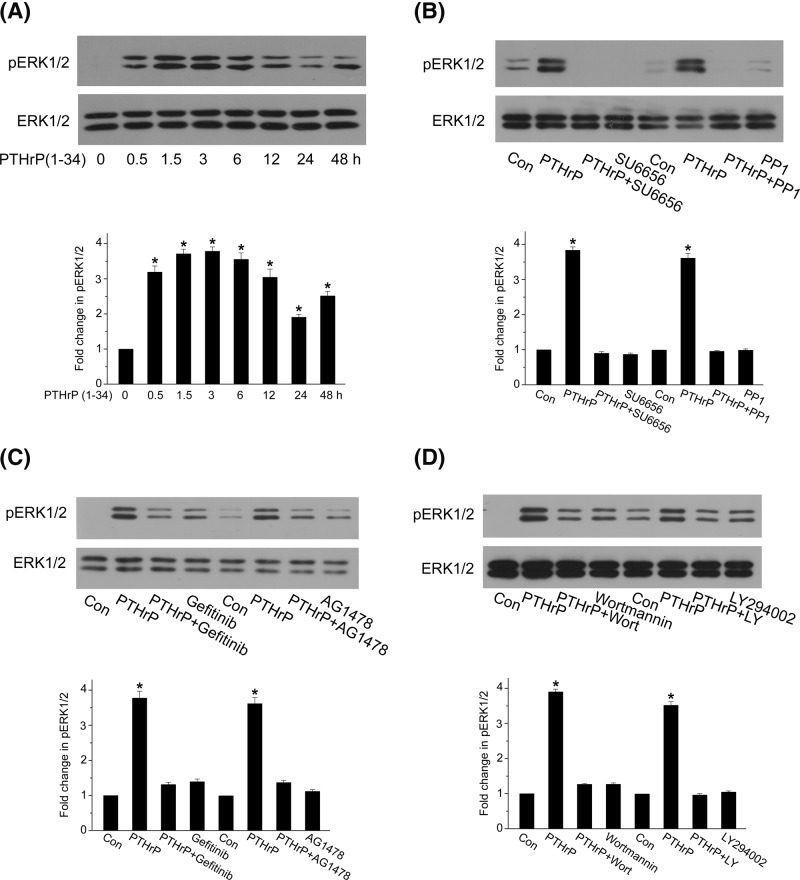
PTHrP-induced ERK1/2 activation is also mediated by Src/EGFR/PI3K signaling **(A**) MCs were treated with 100 nM PTHrP (1–34) for indicated duration, and ERK1/2 phosphorylation was assessed by western blot (*P<0.05 vs control, *n*=3). (**B**) MCs were pretreated with Src inhibitor, PP1 or SU6656, prior to PTHrP (1–34) incubation for 1 h. ERK1/2 phosphorylation was assessed by western blot (*P<0.05 vs control, *n*=3). (**C**) MCs were pretreated with EGFR inhibitor, AG1478 or Gefitinib, before PTHrP (1–34) treatment for 1 h. ERK phosphorylation was assessed by western blot (*P<0.05 vs control, *n*=4). (**D**) MCs were pretreated with PI3K inhibitor, Wortmannin or LY294002, prior to PTHrP (1–34) treatment for 1 h. ERK1/2 phosphorylation was detected by western blot (*P<0.05 vs control, *n*=4).

**Figure 8:**
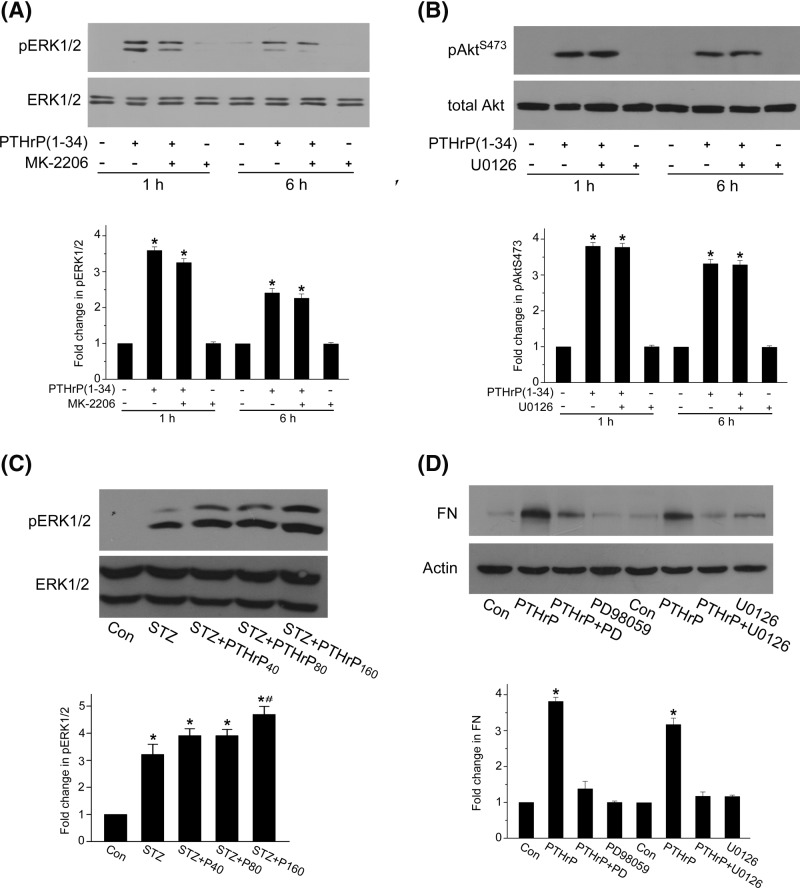
Akt and MEK/ERK1/2 execute parallel signaling pathways that increase fibronectin expression **(A**) MCs were pretreated with MK-2206, a specific Akt kinase inhibitor, prior to PTHrP (1–34) incubation for 1 h. ERK1/2 phosphorylation was detected by western blot (*P<0.05 vs control, *n*=4). (**B**) MCs were pretreated with U0126, a specific MEK inhibitor, prior to PTHrP (1–34) treatment for 1 h. Akt phosphorylation at S473 was assessed by western blot (*P<0.05 vs control, *n*=3). (**C**) Diabetic SD rats were untreated or treated with PTHrP (1–34) by s.c. injection at 40, 80, or 160 μg/kg body weight for 3 months. The phosphorylation level of ERK1/2 in renal cortex was detected by western blot (*P<0.05 vs control, ^#^*P*<0.05 vs STZ group, *n*=8). (**D**) MCs were pretreated with specific MEK inhibitor, PD98059 or U0126, followed by PTHrP (1–34) incubation for 24 h. The protein level of fibronectin was assessed by western blot, with β-actin used as loading control (*P<0.05 vs control, *n*=4).

ERK1/2 is often an effector for EGFR signaling [20,31], and we also checked ERK1/2 activity in rat MCs. From Figure 7A, it is clear that PTH (1–34) induced a strong activation of ERK1/2 detectable as ERK1/2 phosphorylation, which increased within 30 min and continued to 48 h. Both Src inhibitors and EGFR kinase inhibitors efficiently prevented ERK1/2 phosphorylation (Figure 7B,C). These results suggested that PTHrP (1–34) triggered ERK1/2 activation through a Src and EGFR-dependent mechanism. Interestingly, we found that ERK1/2 phosphorylation can also be markedly inhibited by either LY294002 or wortmannin, another highly specific PI3K inhibitor (Figure 7D), indicating that PTH (1–34)-induced ERK1/2 activation is PI3K dependent. Thus, Src/EGFR/PI3K signals to both Akt and ERK1/2 in response to PTHrP (1–34) in rat MCs. To explore the relationship between Akt and ERK1/2 pathway, we used MK-2206, a specific Akt kinase inhibitor, and U0126, a specific MEK inhibitor. As shown in Figure 8A,B, ERK1/2 phosphorylation by PTH (1–34) is unaffected by MK-2206, at the same time, Akt phosphorylation at S473 is also unaffected by U0126. ERK1/2 phosphorylation increased in 160 μg/kg PTHrP (1–34) group compared with diabetic rats (Figure 8C), and the MEK inhibitors PD98059 and U0126 also inhibit fibronectin up-regulation by PTHrP (1–34) in rat MCs (Figure 8D). These results suggested that Akt and MEK/ERK1/2 are likely to execute parallel signaling pathways that increase fibronectin expression.

## Discussion

In the present study, we found for the first time that PTHrP (1–34) peptide-induced fibronectin up-regulation could be independent of TGF-β/Smad signaling in rat MCs. PTHrP (1–34) exposure induces ROS-dependent, Src kinase-mediated EGFR transactivation and subsequent Akt and ERK1/2 activation, which lead to increased fibronectin expression. In STZ-induced diabetic rat, long-term treatment of PTHrP (1–34) peptide was found to result in increased renal hypertrophy, higher expression of p47^phox^, phosphorylation of EGFR, Akt and ERK1/2, and higher expression of fibronectin in renal cortex.

TGF-β/Smad signaling has been recognized as a key mediator in ECM accumulation and renal fibrosis [32,33]. PTHrP might promote EMT through interaction with TGFβ1 in renal tubule cells [8,9]. Our previous study showed that PTHrP could induce fibronectin up-regulation in rat MCs [13]. In the present work, we tested the role of TGF-β1 and found that although PTHrP (1–34) peptide leads to increased TGF-β1 and TβRII expression in rat MCs, PTHrP (1–34) induced-fibronectin up-regulation could be independent of TGFβ/Smad signaling.

Activation of PTH1R results in multiple signaling events, including activation of cAMP/PKA, phospholipase C/PKC [17], EGFR [9,19], and ERK1/2 [20,31]. We next evaluated the involvement of these protein kinases in fibronectin up-regulation induced by PTHrP treatment in rat MCs. Our results indicated that PTHrP (1–34)-mediated fibronectin up-regulation is PKA and PKC independent. PTHrP (1–34) induced persistent EGFR transactivation, as determined by tyrosine phosphorylation at Y845 rather than Y1173, which is phosphorylated by autophosphorylation after EGF-like ligand-mediated activation such as HB-EGF. Furthermore, both MMPs inhibitor and the HB-EGF inhibitor could not prevent EGFR phosphorylation at Y845, indicating that PTHrP (1–34) can transactivate EGFR by a nonligand-dependent way. It has been reported that EGFR can be directly phosphorylated at Y845 by Src kinase [34–36]. We thus explored the role of Src in PTHrP (1–34)-induced EGFR transactivation and found that EGFR phosphorylation at Y845 requires Src kinase activity.

Src is known to be sensitive to oxidative stress [37,38]. NOX-derived ROS mediate Src activation in different cell types [24–26]. Our study showed that PTHrP (1–34) increased ROS production quickly in rat MCs, which can be inhibited by NOX inhibitor, apocynin. Both apocynin and antioxidant NAC inhibited Src activation and EGFR phosphorylation at Y845. Put together, these results indicated that PTHrP (1–34) induces NOX-dependent ROS generation, which is required for Src and EGFR activation.

PI3K/Akt and MEK/ERK are usually thought to carry out parallel signaling pathways [39]. There is evidence that connective tissue growth factor (CTGF)-induced fibronectin expression in MCs is mediated by activation of independent PI3K/Akt and ERK1/2 pathway, most probably in a Src dependent fashion [40]. However, there are some exceptions. For instance, advanced glycation end products (AGEs)’s receptor agonist, calgranulin S 100, can activate ERK by PI3K-dependent manner in MCs [41]. Similarly, our results indicated that PTHrP (1–34)-induced ERK1/2 and Akt activation require prior PI3K activation, which is dependent on ROS production and subsequent Src-mediated EGFR activation. Moreover, MEK inhibitor cannot inhibit Akt phosphorylation and vice versa, suggesting that as the downstream effectors of PI3K, Akt and ERK1/2 might parallelly lead to excessive protein synthesis of fibronectin.

Although Akt and ERK1/2 signaling pathways are critical in the modulation of ECM expression in MCs and DKD [40–42], the exact mechanisms of Akt and ERK1/2-mediated ECM up-regulation are still under investigation. Recent evidence suggests that PI3K/Akt/mTOR signaling pathway has been considered in the mRNA translation that plays a pivotal role in ECM proteins synthesis in DKD [43,44]. It has been reported that ERK is required for serotonin-induced TGFβ expression in MCs [45]. PI3K, Akt, and ERK signaling pathway have been implicated in the H_2_O_2_-induced TGFβ1 expression [46]. Given that TGFβ1 is up-regulated until 24 h in MCs stimulated by PTHrP (1–34), Akt and ERK1/2 possibly mediate TGFβ1 up-regulation. Smad2/3 phosphorylation did not increase at 72 h of PTHrP (1–34) treatment, indicating that TGFβ1-dependent ECM up-regulation might appear later.

In order to further explore the role of PTHrP (1–34) in DKD, STZ-induced diabetic rats were established. We found that PTHrP (1–34) injection did not change serum calcium and phosphate levels, which is consistent with previous studies [16,47,48], indicating that up to 160 μg/kg PTHrP (1–34) does not cause hypercalcemia. Although PTHrP (1–34) aggravated renal hypertrophy in diabetic rats, there is no difference of UAE between diabetic rats and PTHrP (1–34) groups. It has been reported that PTHrP transgenic mouse developed increased renal hypertrophy and a higher UAE in STZ-induced experimental DKD [7]. The difference of our results may be explained by several reasons: (1) PTHrP was administered systemically in SD rats rather than transgenic overexpression in the renal proximal tubule; (2) PTHrP was administered intermittently rather than continuous expression; and (3) PTHrP (1–34) amino-terminal peptide was used rather than full-length PTHrP.

Growing evidence demonstrated that oxidative stress plays an important role in the pathogenesis of DKD [49]. Major ROS generators in the glomerulus of the kidney are NOX1 and NOX2 [27,28]. The activation of NOX1 and NOX2 is dependent on several cytosolic regulatory subunits, including p47^phox^ [29,30]. In cellular experiments, we showed that NOX-dependent ROS generation is required for PTHrP (1–34)-induced Src activation and EGFR phosphorylation. Thus, we investigated the role of PTHrP (1–34) in the expression of p47^phox^ and found an increased p47^phox^ protein level in PTHrP (1–34)-treated diabetic rat kidney. The phosphorylation level of EGFR, Akt and ERK1/2 was also higher in the kidney cortex of diabetic rats that treated by PTHrP (1–34). Moreover, increased fibronectin protein level in diabetic kidney was observed in PTHrP (1–34)-injected rats. Put together, it is feasible to consider that chronic PTHrP (1–34) administration might exacerbate oxidative stress, subsequently activate EGFR, Akt and ERK1/2 signaling, and eventually lead to ECM accumulation in diabetic rat kidneys.

**Figure 9:**
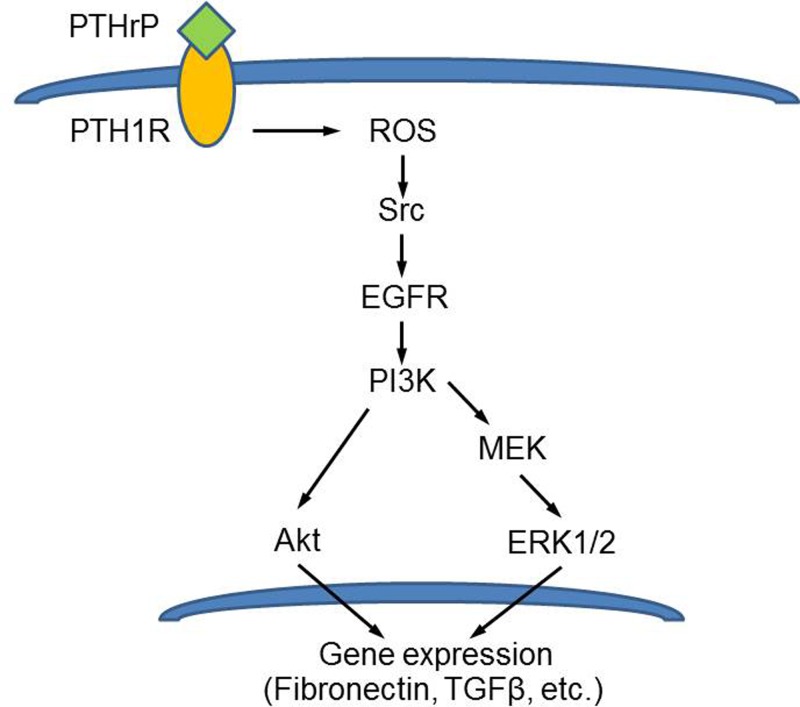
Schematic of PTHrP (1–34)-induced signaling cascade in rat MCs PTHrP (1–34) leads to NOX-dependent ROS production, Src activation, and subsequent EGFR transactivation. Downstream of Akt S473 phosphorylation and MEK/ERK1/2 activation requires EGFR-mediated PI3K activation. Akt and ERK1/2 possibly execute parallel signaling pathways that increase fibronectin and TGF-β expression.

As summarized in Figure 9, the present study demonstrates that NOX-derived ROS mediates Src-dependent EGFR transactivation and PI3K activation induced by PTHrP (1–34) in rat MCs. As the downstream effectors of PI3K, Akt and ERK1/2 discretely lead to excessive protein synthesis of fibronectin. Current data bring some new insights into the complex mechanisms of PTHrP as a factor involved in the progression of DKD and provide new thought for the therapeutic strategy of glomerular sclerosis.

## Supporting information

**Supplementary Figure 1:** PTHrP (1-34)-induced fibronectin upregulation is independent of Smad2/3, PKA and PKC signaling.

**Supplementary Figure 2:** NADPH oxidase inhibitor apocynin prevented PTHrP (1-34)-induced hydrogen peroxide generation.

**Supplementary Figure 3:** Apocynin and NAC inhibited PTHrP (1-34)-induced superoxide generation.

## Abbreviations

BSA: bovine serum albumin
DAB: 3, 3′-diaminobenzidine
DCFH-DA: 2′,7′-dichlorodihydrofluorescin diacetate
DKD: diabetic kidney disease
ECM: extracellular matrix
EGF: epidermal growth factor
EGFR: epidermal growth factor receptor
EMT: epithelial-mesenchymal transition
MC: mesangial cell
MMP: matrix metalloproteinase
NADPH: nicotinamide adenine dinucleotide phosphate
NOX: NADPH oxidase
PTH: parathyroid hormone
PTHrP: parathyroid hormone-related protein
ROS: reactive oxygen species
RT-PCR: quantitative real-time PCR
SOD: superoxide dismutase
STZ: streptozotocin
UAE: urinary albumin excretion
VEGF: vascular endothelial growth factor
