# Supplementary material for: Parathyroid hormone-related protein induces fibronectin up-regulation in rat mesangial cells through reactive oxygen species/Src/EGFR signaling
Source: Biosci Rep. 2019 Apr 26;39(4):BSR20182293. doi: 10.1042/BSR20182293 (PMC6487264; doi:10.1042/BSR20182293)
Supplement: Supplementary file 1 [file bsr20182293_Supp1.pdf]

Supplementary Figure Legends

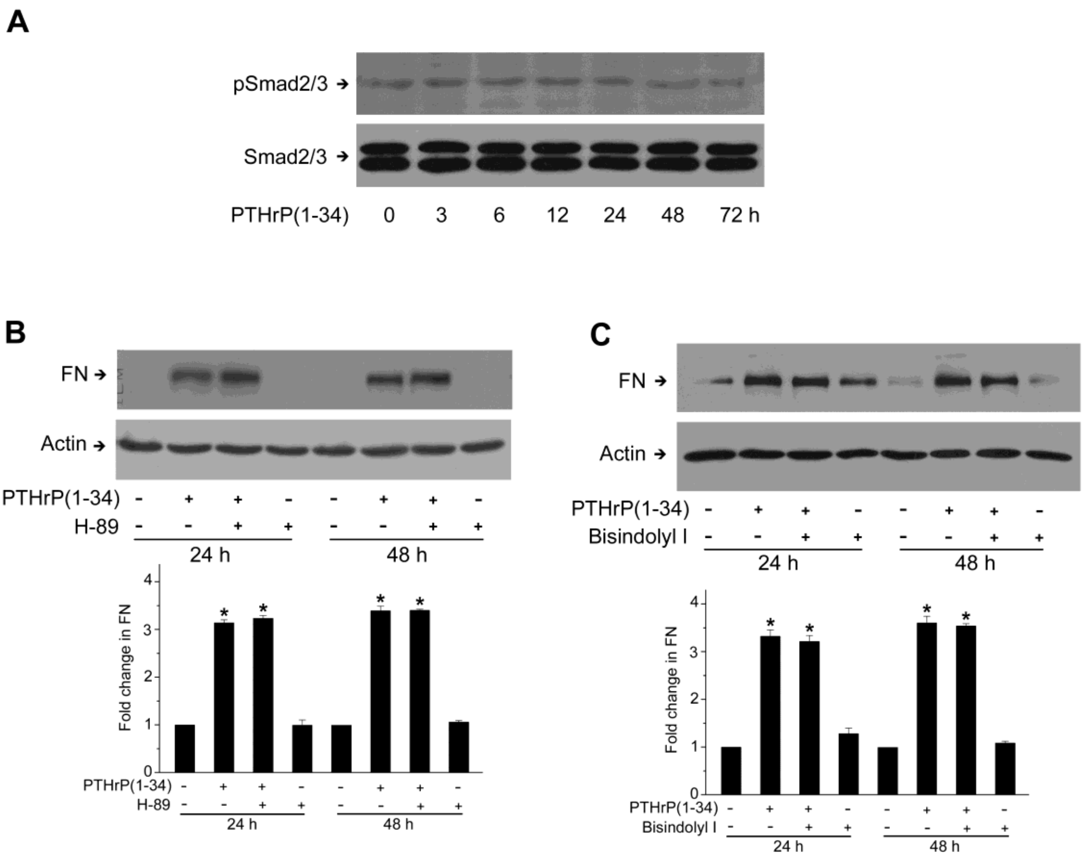

Fig. 1 PTHrP (1-34)-induced fibronectin upregulation is independent of Smad2/3, PKA and PKC signaling. A: MCs were treated with 100 nM PTHrP (1-34) for indicated time. Phosphorylation of Smad2/3 was detected by Western blot. B, C: MCs were pretreated with the PKA inhibitor H-89 (B) or the PKC inhibitor Bisindolylmaleimide I (C) prior to PTHrP (1-34) incubation for 24 or 48 hours. Protein level of fibronectin was assessed by Western blot (*$p < 0.05$ vs. control, n = 3).

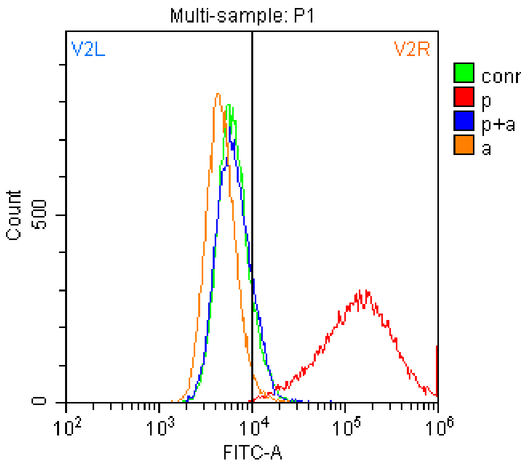

Fig. 2 NADPH oxidase inhibitor apocynin prevented PTHrP (1-34)-induced hydrogen peroxide generation. MCs were incubated with DCFH-DA at 37 °C for 30 min, and then treated or untreated with selective NADPH oxidase inhibitor, apocynin, before 100 nM PTHrP (1-34) stimulation for 5 min. Hydrogen peroxide (H_2O_2) production was assayed by flow cytometry and representative graph of DCFH-DA fluorescence intensity (excitation wavelength 488 nm and emission wavelength 535 nm) was shown.

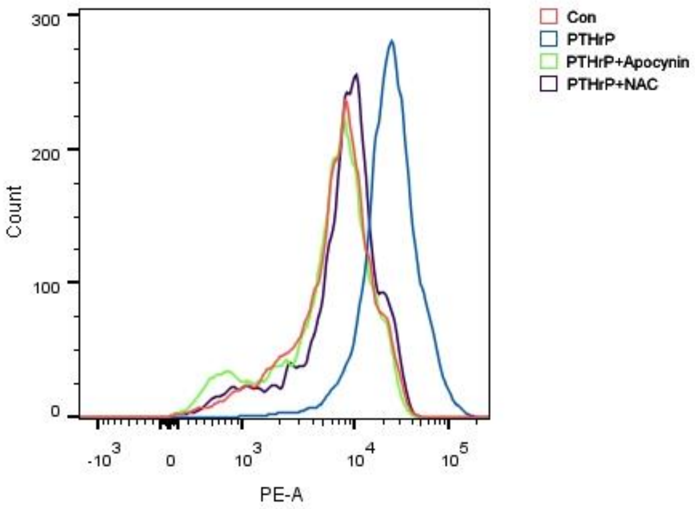

Fig. 3 Apocynin and NAC inhibited PTHrP (1-34)-induced superoxide generation. MCs were incubated with DHE at 37 °C for 30 min, and then treated with apocynin or NAC before 100 nM PTHrP (1-34) stimulation for 5 min. When DHE is ingested by living cells, it can dehydrogenate to produce ethidium under the action of superoxide anion in cells. Samples were analyzed by flow cytometry and representative graph of ethidium fluorescence intensity (excitation wavelength 300 nm and emission wavelength 610 nm) was shown.
